# Induction of apoptosis and autophagy via mitochondria- and PI3K/Akt/mTOR-mediated pathways by *E. adenophorum* in hepatocytes of saanen goat

**DOI:** 10.18632/oncotarget.10402

**Published:** 2016-07-05

**Authors:** Yajun He, Quan Mo, Biao Luo, Yan Qiao, Ruiguang Xu, Zhicai Zuo, Junliang Deng, Xiang Nong, Guangneng Peng, Wei He, Yahui Wei, Yanchun Hu

**Affiliations:** ^1^ Key Laboratory of Animal Disease and Human Health of Sichuan Province, College of Veterinary Medicine, Sichuan Agricultural University, Sichuan Province, Wenjiang 611130, China; ^2^ College of Life Science, Leshan Normal University, Le'shan, 614000, China; ^3^ Key Laboratory of Resource Biology and Biotechnology in Western China, School of Life Science, Northwest University, Xi'an 710069, China

**Keywords:** E. adenophorum, apoptosis, autophagy, mitochondrial and PI3K/Akt/mTOR pathway, hepatocytes

## Abstract

*E. adenophorum* has reported to cause hepatotoxicity. But, the precise effects of *E. adenophorum* on hepatocytes is unclear. Saanen goats were fed on *E. adenophorum* to detect the cytotoxicity effects of *E. adenophorum* on hepatocytes. Our study has shown that the typical apoptotic features, the increasing apoptotic hepatocytes and activated caspase-9, −3 and the subsequent cleavage of PARP indicated the potent pro-apoptotic effects of *E. adenophorum*. Moreover, the translocation of Bax and Cyt c between mitochondria and cytosol triggering the forming of apoptosome proved that the mitochondria-mediated apoptosis was triggered by *E. adenophorum*. Furthermore, *E. adenophorum* increased the MDC-positive autophagic vacuoles and the subcellular localization of punctate LC3, the ratio of LC3-II/LC3-I and the protein levels of Beclin 1, but decreased that of P62, indicating the potent pro-autophagic effects of *E. adenophorum*. In addition, *E. adenophorum* significantly inhibited the protein leves of p-PI3K, p-Akt and p-mTORC1, but increased PTEN and p-AMPK. Also, the p-mTORC2 and p-Akt Ser473 were inhibited, indicating that the supression of mTORC2/Akt pathway could induce the autophagy of hepatocytes. The autophagy-realted results indicated that the inhibition of PI3K/Akt/mTORC1- and mTORC2/Akt-mediated pathways contributed to the pro-autophagic activity of *E. adenophorum*. These findings provide new insights to understand the mechanisms involved in *E. adenophorum*-caused hepatotoxicity of Saanen goat.

## INTRODUCTION

*Eupatorium adenophorum* (*E. adenophorum*) is a perennial herbaceous plant that is highly adaptable and known as the important destructive exotic species. Nowadays *E. adenophorum* has been the global pest of crops and forests and became the major weed of natural environments, forests, crops and plantations, which causes environmental and ecological damages in numerous places worldwide [1–4]. At present, many aspects of *E.adenophorum* are being studied widely [5]. Previous study indicated that *E. adenophorum* has the acaricidal activity [6–8], antitumor activity [9, 10], potential anti-inflammatory and other biological activities [11]. The previous reports showed that purified extracts from *E. adenophorum* could cause hepatotoxicity of mice and livestock [8, 12–15], especially the 9-oxo-10,11-dehydro-agerophorone which is the main poisonous components of *E. adenophorum* [16, 17]. Also, when administrated with *E. adenophorum* freeze-dried leaf powder, the mice were caused hepatotoxicity. It's reported that methanolic extract of *E. adenophorum* collected from India can cause albino mice hepatotoxicity. Moreover, horses administrated with *E. adenophorum* could be caused pulmonary toxicity and chronic pulmonary disease [18]. Liver plays the centrol role in drug metabolism and is responsible for substances modulating biotransformation [19]. Autophagy occurs to eliminate the damaged organelles and protein aggregates in cell level, aiming at maintaining the cytoplasmic homeostasis [20]. Apoptosis, an essential physiological process, is a pattern of cell death during various physiological and pathological conditions [21, 22], while toxic stimuli promote apoptosis process [23].

*E*. *adenophorum*-related researches indicated that it might be as the potential inducer in inhibitting growth and inducing apoptosis and autophagy in hepatocytes by causing hepatotoxicity. Our present study was conducted to investigate the cytotoxicity effects of *E*. *adenophorum* on hepatocytes, aiming at explicating the possible mechanisms involved in *E*. *adenophorum*-caused hepatotoxicity of goat.

## RESULTS

### The morphological observation of autophagy and apoptotic hepatocytes

**Figure 1:**
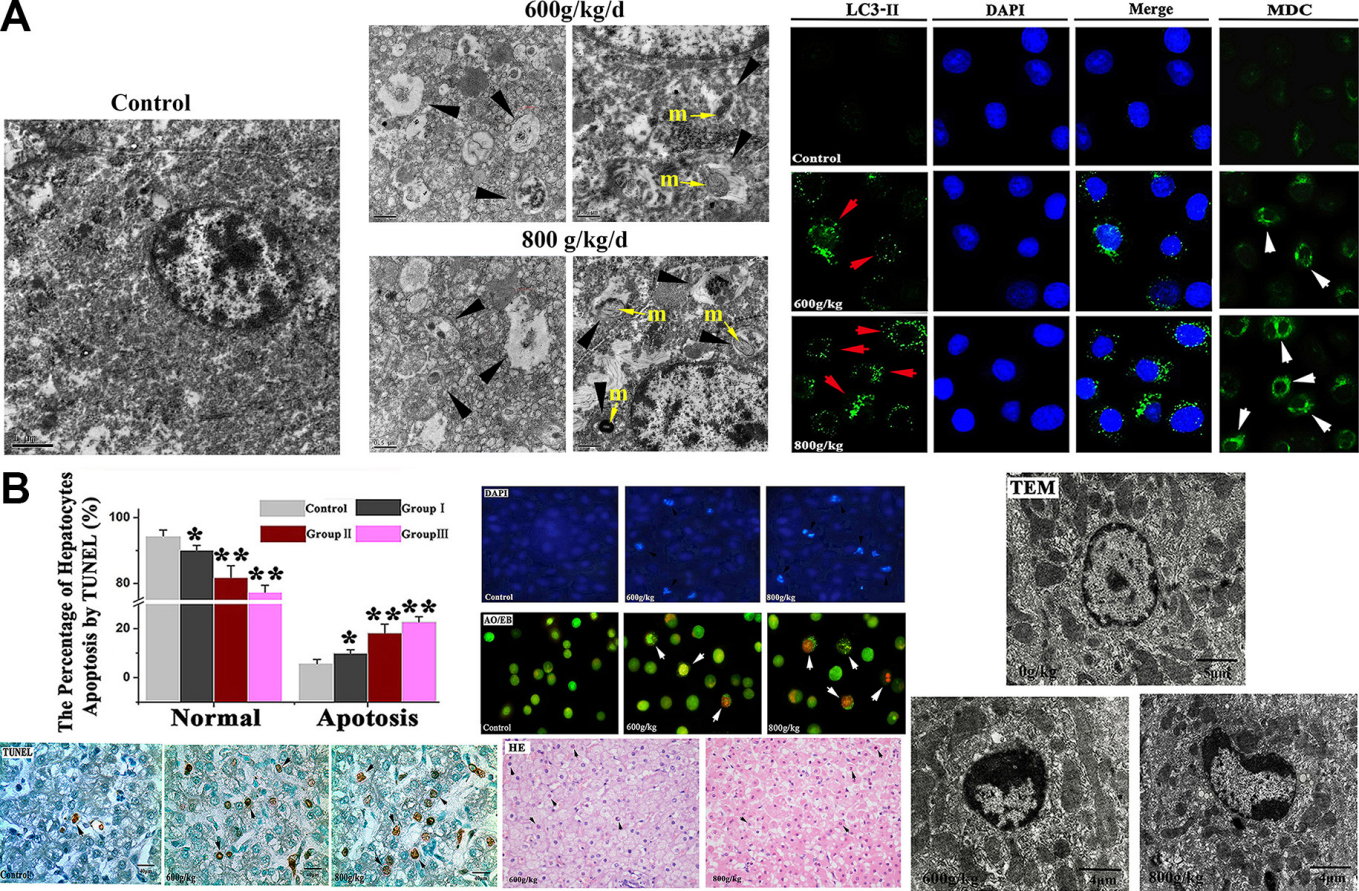
The Morphological changes of autophagy and apoptotic hepatocytes induced by *E. adenophorum* (A) Morphological observation of autophagy in hepatocytes. The characteristic ultrastructural morphology of autophagy in hepatocytes such as autophagic vacuoles (black arrowheads) and double-membrane vesicles formed around pieces of mitochondrion (yellow arrow), ×12000. m, mitochondrion. Hepatocytes were stained with MDC (white arrows) and LC3- II (red arrows) antibody, respectively. Nuclei were stained with DAPI (blue) (bar: 10 μm). (B) The Detection of apoptotic hepatocytes by TUNEL, DAPI, AO/EB, TEM and HE. The number of TUNEL positive cells (indicated arrows) was counted from five random microscopic fields, magnification, ×1000. Representative liver sections were analyzed using DAPI and AO/EBfor apoptotic cell death. Nuclear morphological changes in hepatocytes were observed under fluorescent microscope after DAPI staining (indicated arrows, 200×) and AO/EB staining (200×). The Photomicrographs of liver section and ultrastructural morphology changes of hepatocytes were conducted with HE staining and TEM. Normal cells have an intact membrane, organelles, and normal nuclear morphology. Apoptotic cells show homogeneous chromatin condensation within the nucleus (blace arrows). The condensed chromatin in the apoptotic cell is localized to the periphery of the nucleus. Membranes, cytoplasm, and organelles are intact. Magnification, ×10000. The liver section stained hematoxylin and eosin(HE). The apoptotic hepatocytes showed chromatin condensation and the condensed chromatin was localized to one side of the nucleus (blace arrows). Magnification, ×400.

DAPI, MDC and LC3 immunostaining using fluorescent antibodies to LC3-II were conducteded to confirm autophagy induction by *E. adenophorum*. Also, different sections of livers were measured by TEM to detect apoptosis and autophagy respectively. In experimental groups, characteristic ultrastructural morphology of autophagy was also observed. when the hepatocytes were viewed by TEM, the mitochondrial fragmentation were observed and sections of the mitochondrion appeared to be surrounded by double-membrane profiles. The results of LC3-II immunostaining indicated that *E. adenophorum* increased subcellular localization of punctate LC3-II and the LC3-II puncta formation was increased in hepatocytes. Moreover, the hepatocytes of control showed diffused MDC-staining, whereas *E. adenophorum* increased the fluorescence intensity of hepatocytes indicating extensive MDC-positive autophagic vacuoles. The data above definitely showed that abundant autophagic vacuoles could be induced by *E. adenophorum* (Figure 1A). The TUNEL was performed to study DNA fragmentation in hepatocytes. After mounting the TUNEL positive cells, TUNEL assay showed evident apoptosis in liver sections on *E. adenophorum*-treated condition and the quantification indicated a 18.22% and 22.70% amount of TUNEL positive cells in the group II, III. Then, AO/EB staining and DAPI staining were conducted and showed that *E. adenophorum*-administrated hepatocytes displayed different degrees of chromatin condensation, cell membrane integrity destruction and fragmentation of nuclear. The observation of TEM characteristically showed that hepatocytes displayed morphological changes. The apoptotic hepatocytes’ plasma membrane remains intact and some apoptotic cells featuring decreased cell size and condensation of nuclear chromatin were found. Chromatin condenses against the nuclear membrane, which produces the crescentic pattern chromatin at the early stage of apoptosis. Then chromatin condenses into solid androunds masses, then the solid chromatin will undergo fragmentation (black arrowheads). Histopathologically, liver section stained hematoxylin and eosin (HE). The apoptotic hepatocytes showed chromatin condensation and the condensed chromatin was localized to one side of the nucleus (×400) (Figure 1B).

### The detection of apoptotic hepatocytes

**Figure 2:**
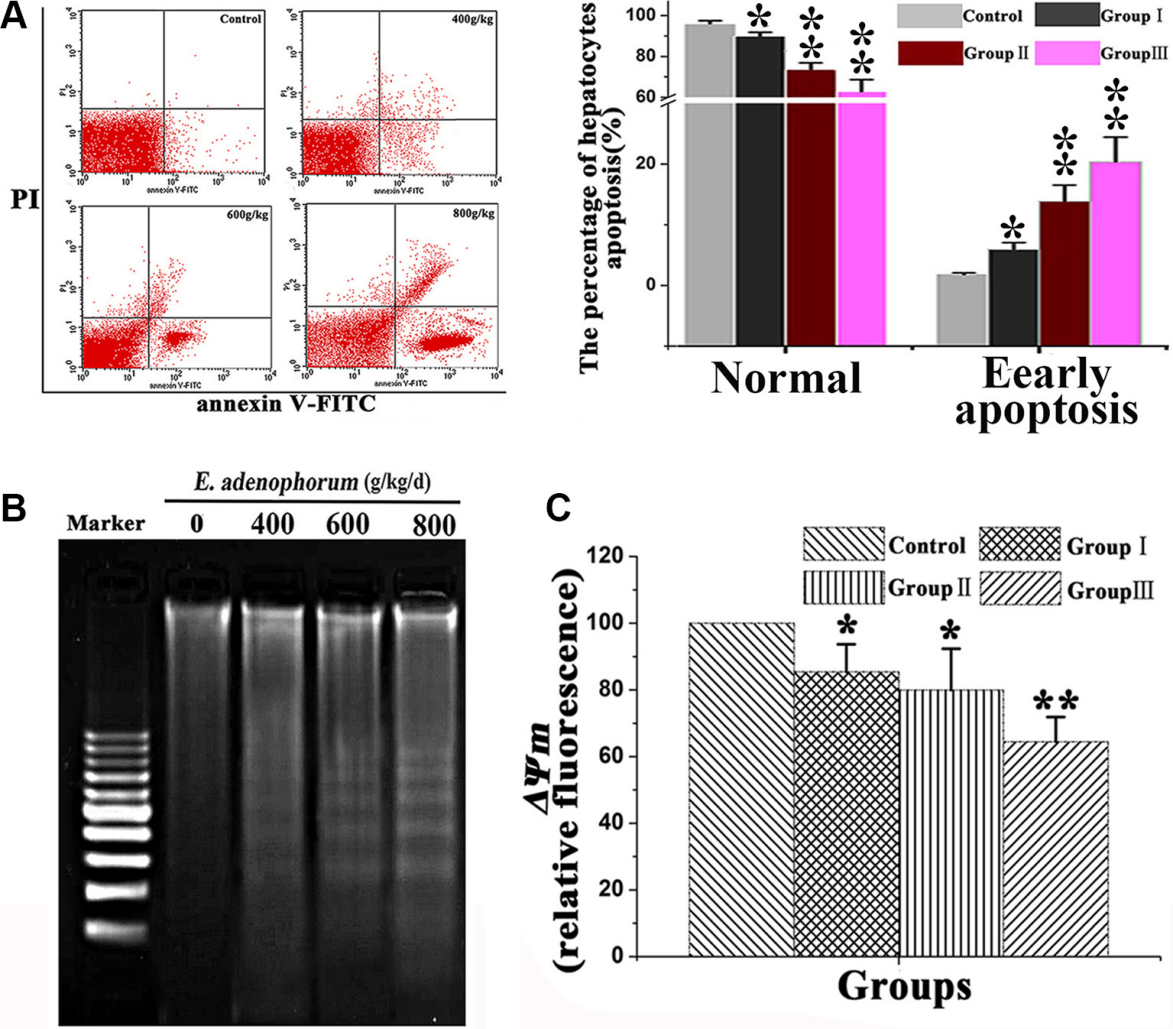
*E. adenophorum* administration induces apoptosis in hepatocytes (**A**) The scattergram of apoptotic hepatocytes. The hepatocytes were analyzed by flow cytometry for Annexin V and PI staining. *E. adenophorum* significantly induced apoptosis in hepatocytes. (**B**) Induction of DNA fragmentation. DNA isolated from hepatocytes was subjected to 2% agarose gel electrophoresis, followed by visualization of bands and photography. (**C**) Flow cytometry and JC-1 measure the effet of *E. adenophorum* on *ΔΨm*. Data are presented with the means ± SD and mean values of three independent experiments. *p < 0.05, compared with the control group; **p < 0.01, compared with the control group.

The apoptotic hepatocytes was further detected via FCM which indicated that *E. adenophorum* inhibited hepatocytes growth accompanied by increasing the proportion of apoptotic hepatocytes. Moreover, the percentage of normal hepatocytes was decreased markedly (Figure 2A). Under agarose gel electrophoresis, We observed the formation of a typical DNA ladder which indicated the apoptotic DNA fragmentation evident in experimental groups after *E. adenophorum*-administration, which further confirmed the induction of apoptosis (Figure 2B). Then we subsequently assessed the apoptosis induced by *E. adenophorum* via evaluating the changes of *Δψm* affected by *E. adenophorum*. The results indicated that the *ΔΨm* significantly decreased which was measured by FCM (Figure 2C). The results demonstrated that *E. adenophorum* inhibits hepatocytes growth by inducing hepatocytes apoptosis.

### The autophagy and apoptosis were triggered by *E. adenophorum*

**Figure 3:**
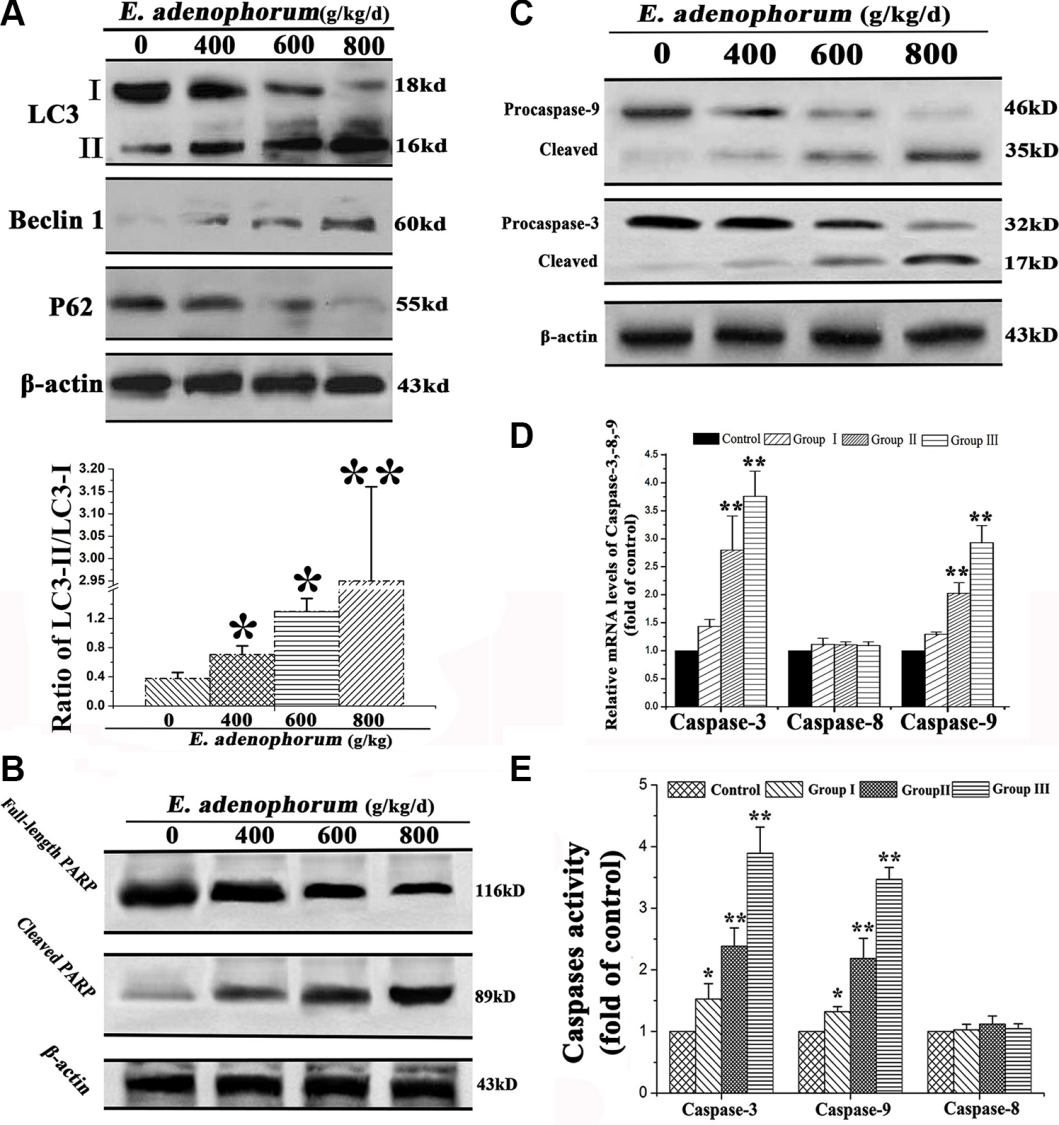
The autophagy and apoptosis were activated by *E. adenophorum* (**A**) The autophagy was triggered by *E. adenophorum* in hepatocytes. The representative blots show the expression levels of LC3-I, LC3-II, Beclin 1 and p62 in hepatocytes treated with *E. adenophorum*. β-actin was used as the internal control. Bar graph shows the ratio of LC3-II/LC3-I. (**B**) *E. adenophorum* activated apoptosis of hepatocytes. The hepatocytes were subjected to western blot analysis to detect full-length and cleaved PARP. (**C**) The protein levels of procaspase-3, -9 and the cleaved form of them which were shown with β-actin as a control were detected by Western-blot analysis. (**D**) The total mRNA was extraced and the relative mRNA levels of caspase-3, -8 and -9 were detected through qRT-PCR assay. (**E**) Caspase activities in *E. adenophorum*-treated hepatocytes. BCA assay was used to equal protein amounts and the enzymatic activities of caspases-8, -9, and -3 were measured using the colorimetric assay kits. All data are presented with the means ± SD and mean values of three independent experiments. *p < 0.05, compared with the control group; **p < 0.01, compared with the control group.

To confirm the formation of autophagosome in hepatocytes, immunoblotting was performed to measure the changes of three major autophagy-related markers (i.e. LC3, Beclin-1 and P62). Beclin 1 and LC3-II protein levels were remarkably increased, but the levels of LC3-I and P62 were significantly decreased (Figure 3A). Then, we conducted next study to investigate the potential mechanisms of *E. adenophorum*-induced apoptosis in hepatocytes. PARP, an indicator of apoptosis, was appeared to be cleaved from 116 to 85 kDa fragments (Figure 3B). Caspase is known to play a key role in numerous apoptosis-mediated signaling [24]. Then the activity of initiator caspase-8, -9 and effector caspase-3 [25, 26] were measured via western blot. As presented in Figure 3C, the *E. adenophorum*-administration led to increased activated forms of caspase-9, -3 in hepatocytes. *E. adenophorum* markedly activated the relative mRNA levels of caspases-9, -3, but failed to induce the caspase-8 (Figure 3D). To further determine that caspases were involved in *E. adenophorum*-induced apoptosis, the colorimetric assay kits was performed to measure the activities of caspase-8, -9, and -3. The results showed that *E. adenophorum* induced the activation of caspases-9, -3, but not caspase-8 (Figure 3E). Together, these data showed that *E. adenophorum* activated autophagy and apoptosis in hepatocytes.

### The mitochondrial apoptotic pathway was activated by *E. adenophorum*

**Figure 4:**
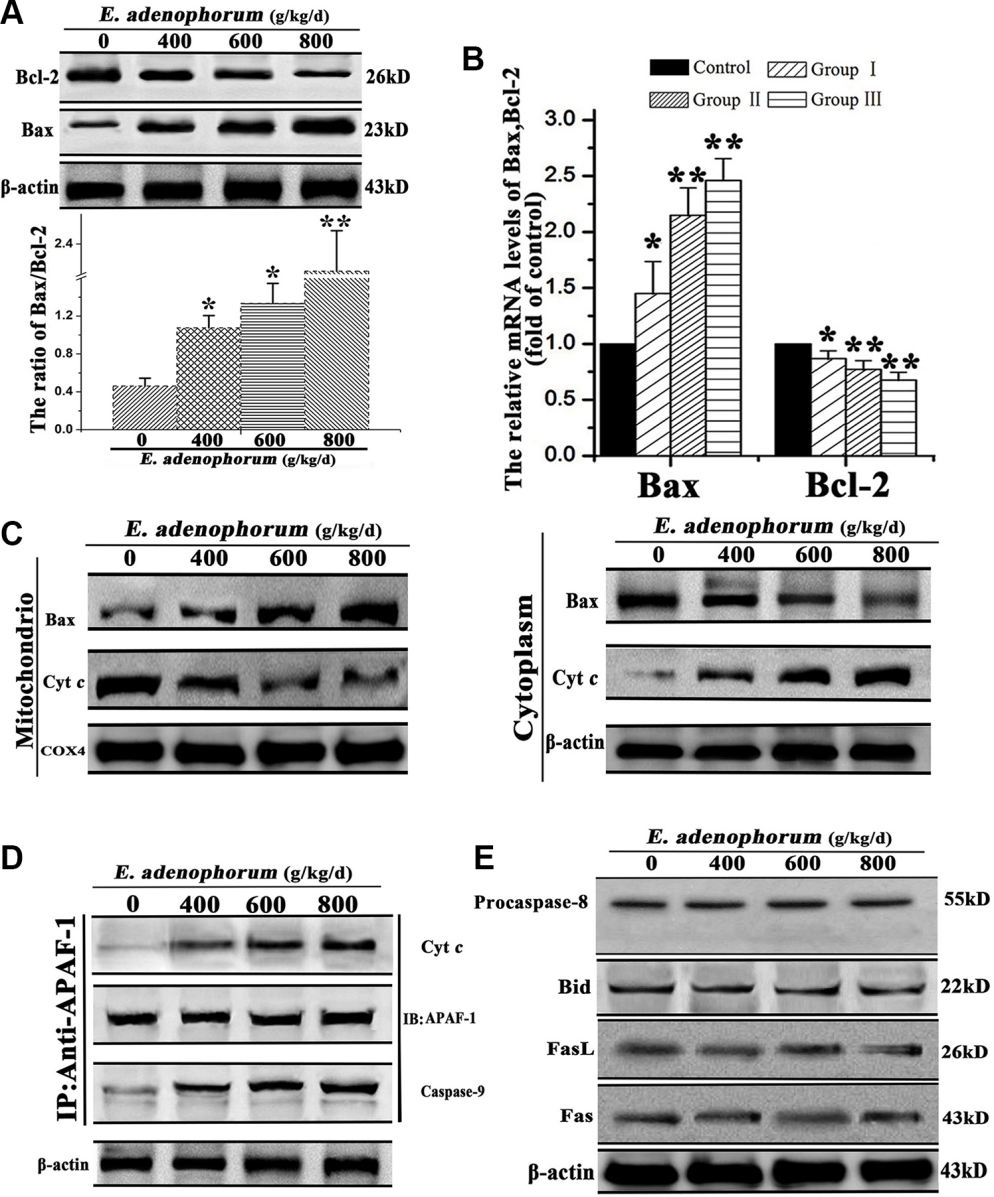
The mitochondria-mediated apoptosis of hepatocytes was triggered by *E. adenophorum* (**A**) *E. adenophorum* increased the protein level of Bax but decreased that of Bcl-2 detedted through western blot resulting in the increasing ratio of Bax/Bcl-2. (**B**) The total mRNA was extraced and the relative mRNA levels of Bax and Bcl-2 were detected through qRT-PCR assay. All data are presented with the means ± SD and mean values of three independent experiments. *p < 0.05, compared with the control group; **p < 0.01, compared with the control group. (**C**) E. adenophorum induced Bax translocation and Cyt c release. The cytosolic and mitochondrial fraction proteins were collected and then detected by western blot. COX 4 and β-actin were used as internal controls for the mitochondrial fractions and the cytosolic fraction, respectively. (**D**) E. adenophorum induced apoptosome formation. Protein extractions from hepatocytes were collected and used in immunoprecipitation assays against Apaf-1. The levels of caspase-9 and Cyt c were detected by western blot to indicate the formation of apoptosome complex. (**E**) Representative blots show the expression levels of procaspase-8, caspase-8, Bid, FasL and Fas in hepatocytesadministrated with E. adenophorum for 3 months. β-actin was used as the internal control.

Aiming at clarify the underlying mechanism of *E. adenophorum*-induced apoptotic effect in hepatocytes, we examined the effects of *E. adenophorum* on the pro-apoptotic factor Bax and the anti-apoptotic factor Bcl-2. The results showed that *E. adenophorum* dose-dependently increased the protein and relative mRNA levels of Bax and decreased that of Bcl-2 (Figure 4A and 4B). Then, we measured the release of Cyt *c* and Bax extracted from both mitochondrial and cytosolic fractions. The results proved that a translocation of Bax from cytosol to mitochondria and significant increase release of Cyt *c* from mitochondria to cytosol were observed, which were caused by *E. adenophorum* (Figure 4C). Next, the cell lysates were immunoprecipitated with an anti-Apaf-1 antibody and subsequently subjected to Western blot with anti-caspase-9 and anti-Cyt *c* antibodies in order to detect the apoptosome formation. The results presented that Apaf-1 was interacted with Cyt *c* and caspase-9, indicating the apoptosome formation in hepatocytes (Figure 4D). The results above indicated the activation of the mitochondrial pathway. Previous study illustrated that the death receptors (such as Fas or FasL) can activate the death receptor pathway, and death receptors will recruit fas associated protein with death domain and procaspase-8, resulting in the activation of caspase-8 and the death-inducing signaling complex forming. The activated caspase-8 can cleave Bid to truncated Bid which would translocate to mitochondria [26]. Western blot showed that activated form of procaspase-8 and the protein level of Bid did not show significant variations. Besides, the fail of activating Fas or FasL proved that *E. adenophorum* could not activate the death receptor pathway (Figure 4E). These results indicated that the mitochondrial pathway was activated in *E. adenophorum*-induced apoptosis.

### *E. adenophorum* induces autophagy via the inhibition of PI3K/Akt/mTOR axis

**Figure 5:**
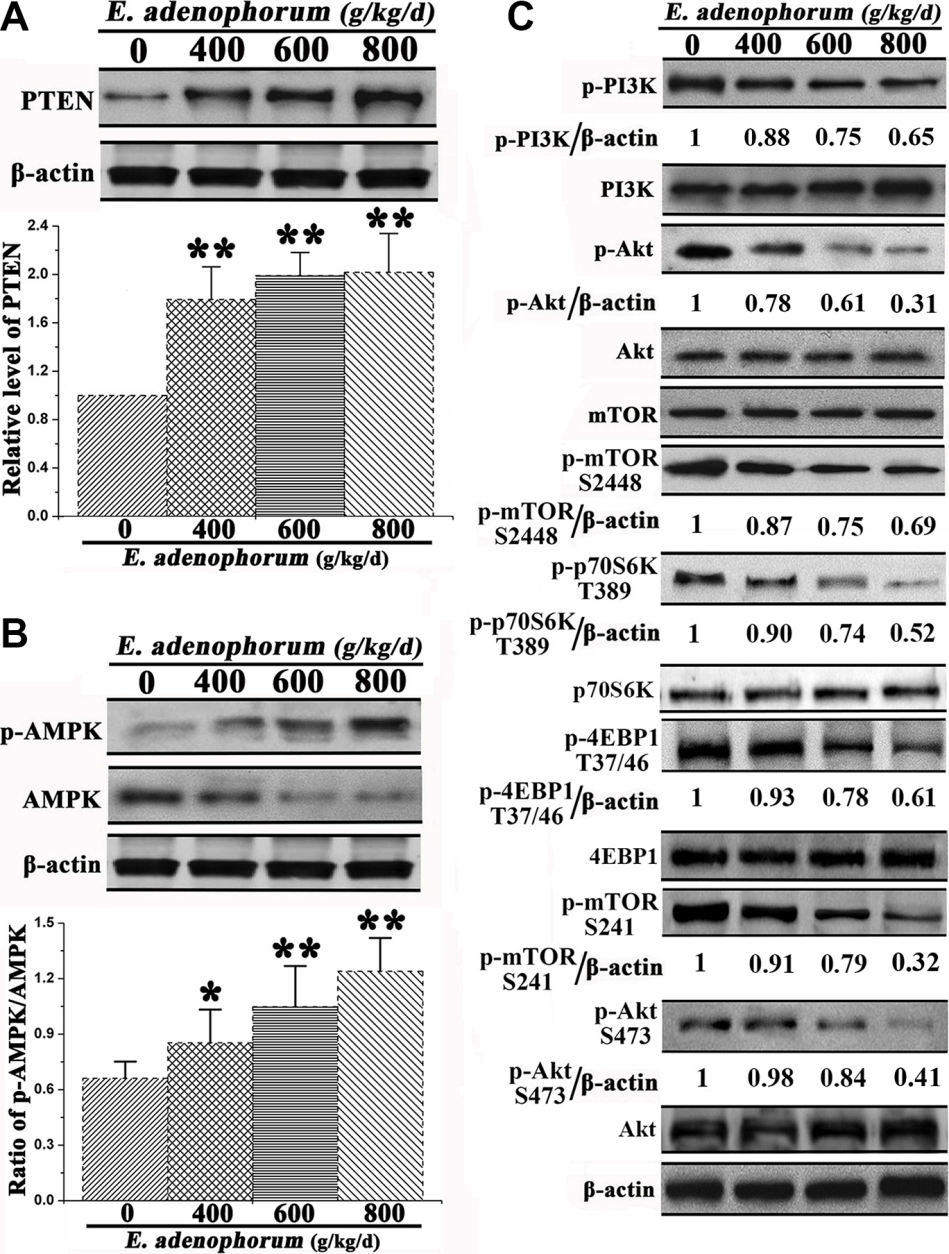
Effect of *E. adenophorum* on PI3K/Akt/mTOR-mediated autophagy signaling pathway (**A**) Protein extractions from hepatocytes were collected and the protein level of PTEN was analysised via western blot. Bar graph shows the relative level of PTEN. (**B**) The phosphorylation level of AMPK and total level of AMPK were analysised by Western-blot. Bar graph shows the ratio of p-AMPK/AMPK. (**C**) Representative blots show the protein levels of PI3K, Akt, and mTOR and the phosphorylation of PI3K, Akt T37/46, mTOR S2448, mTOR S241, p70S60K and 4EBP1 in hepatocytes adminitrated with *E. adenophorum*. Bar graphs showing the ratio of p-PI3K/PI3K, p-Akt/Akt and p-mTOR/mTOR in hepatocytes. All data are presented with the means ± SD and mean values of three independent experiments. *p < 0.05, compared with the control group; **p < 0.01, compared with the control group.

Owing to the observation of the autophagy of hepatocytes induced by *E. adenophorum*, then we explored the underlying mechanisms for the autophagy-inducing effect of *E. adenophorum*. Since PTEN, phosphorylated AMPK and PI3K are the upstream signaling molecules of Akt/mTOR pathway and played a crucial role in the regulation of cell death and proliferation [27]. Then we measured the protein levels of them. The expression level of PTEN which is negative regulator of PI3K/Akt signaling pathway was significantly increased (Figure 5A). AMPK has an important role in the regulation of cell survival and death [28]. *E. adenophorum* increased the level of p-AMPK and reduced the level of AMPK resulting in the increasing ratio of p-AMPK/AMPK (Figure 5B). Then the phosphorylation levels of PI3K which works as the upstream signaling molecules of Akt/mTOR pathway and plays a crucial role in the regulation of cell proliferation and death were explored. *E. adenophorum* inhibited the phosphorylation of PI3K in hepatocytes. Akt is a downstream effector of PI3K, an upstream regulator of mTORC1 and an effector of mTORC2, whereas p70 S6 kinase (P70S6K) and 4E binding protein 1 (4EBP1) are downstream substrates of mTORC1 [29]. There was a siginificant reduction in the level of p-Akt. Yet, the alteration in the expression of Akt measured via western blot showed no significant difference. As shown by recent studies thatmTOR can regulate the cell growth and autophagic cell death and mTORC1 and mTORC2 contain primarily Ser2448 phosphorylation and Ser2481 phosphorylation respectively. Besides, Ser2481 phosphorylation of mTOR presents as the marker of the presence of mTORC2 complexes [29, 30]. We then evaluated the impact of *E. adenophorum* on phosphorylation of mTORC1 and mTORC2 targets. The activity of the mTORC1 pathway was dramatically inhibited by *E. adenophorum*, as indicated by the decreased phosphorylation of mTORC1 and the phosphorylation of its downstream effectors including p70S6K and 4EBP1. In addition, *E. adenophorum* markedly inhibited phosphorylation of mTORC2. Besides, the direct mTORC2 substrate p-Akt Ser473 was markedly inhibited in hepatocytes, resulted in the supression of the mTORC2-Akt signaling pathway (Figure 5C). The above findings exhibited the *E. adenophorum*-induced autophagy effects on hepatocytes through the suppression of the PI3K/Akt/mTOR pathway

## DISCUSSION

Invasion of rangelands by *E. adenophorum* which is an alien species has become a a majar economic and ecological threat in mid-hill regions of the word [1]. The ingestion of *E. adenophorum* was reported to casused hepatotoxicity in mice, anorexia, suspension of rumination and photosensitization in cattle, etc [31]. Previous reports make it possible for exploring *E. adenophorum* as a triggerof hepatotoxicity in saanen goat.

In this study, we have shown that *E. adenophorum* induced hepatotoxicity reflected by apoptotsis and autophagy. In autophagy, Beclin 1 takes part as part of class III PI3k complex and functions as the key factor in the formation of autophagosome. LC3-II plays indispensable roles in autophagosomes formation [32]. Our present results illustrated that *E. adenophorum* activated the autophagy without interfering with autophagic flux in hepatocytes, as evidenced by the changes of p62, LC3-II and Beclin 1 levels. The autophagic vacuoles marker and autophagosomes marker have been respectively reported to be the MDC and LC3 [33]. The extensive MDC-positive autophagic vacuoles and the increased subcellular localization of punctate LC3 definitely showed that *E. adenophorum* induces autophagosome formation in hepatocytes. The PI3K/Akt/mTOR axis is responsible for promoting cell growth, synthesis and metabolism. PI3K activates the Akt, which in turn results in the phosphorylation and activation of mTOR. However, PTEN can inhibit Akt/mTOR signaling [34]. Our present study demonstratedthat *E. adenophorum* promoted the formation of autophagy via the activation of PTEN and AMPK. Since mTORC1 is well known to inhibit autophagy by phosphorylating p70S6K and 4EBP1 [35]. The result suggested that the suppression of mTORC1 signaling pathway may be responsible for the autophagy of hepatocytes. The role of mTORC2 and mTORC2/Akt signaling pathway is reported to have a crucial role in inhibition of the autophagy formation [36], which was similar with our results that the p-mTORC2 and p-Akt Ser473 were inhibited, indicating that the supression of mTORC2/Akt pathway could induce the autophagy of hepatocytes. Thus, our present study demonstrated that *E. adenophorum*-mediated activation of autophagy relies on the PI3K/Akt/mTOR and mTORC2/Akt signaling pathway.

In cell level, the increased apoptotic hepatocytes, the morphological observation of the apoptotic hepatocytes nucleus and the collapse of *ΔΨm* and the DNA fragmentation proved that *E.* adenophorum induced apoptosis and suppressed the growth of hepatocytes. Then the mechanism of *E. adenophorum*-induced apoptosiswas investigated. The death receptor pathway and the mitochondria-mediated pathway are respectively triggered by caspase-8 and caspase-9. The activation of caspase-3 is essential for the development of apoptosis [37]. The release of Cty *c* and mitochondrial integrity is mainly controlled by the Bax and Bcl-2 identified as major regulators [38]. y.Numberous stimulis could converge at the mitochondria in the mitochondria-mediated pathway activating the BH3-only protein family, which would be regulated by members of Bcl-2 family. Such the result would lead to the assembly of Bax within mitochondrial outer membranes, results in the release of Cyt *c* from mitochondria to cytosol. Then, the released Cyt *c* will activate the Apaf-1 to generate apoptosome and tigger caspase 9. The activation of the above process indicates the activation of mitochondrial pathway. Here we showed that *E. adenophorum* decreased the protein and mRNA levels of Bcl-2 and increased that of Bax, and promoted the translocation of Bax from cytoplasm to mitochondria. Besides, the formation of apoptosomes together with procaspase-9, Apaf-1 and Cyt *c* released from dysfunctional mitochondria into cytosol and the activation of caspse-9, -3 and the cleavage of PARP indicated that mitochondria-mediated apoptosis was activated in hepatocytes by *E. adenophorum*. Nevertheless, *E. adenophorum* did not trigger the activation of death receptor pathway-mediated factors caspase-8 and its upstream molecules Fas and FasL and downsream molecule Bid. The data above indicated that *E. adenophorum* is the potential inducer to trigger mitochondria-mediated apoptosis. Bcl-2 and Beclin 1 are invoved in regulating both autophagy and apoptosis. In response to some specific signals, Beclin 1 also works as a vital molecule in the convergence between autophagy and apoptosis. Bcl-2 could inhibit essential autophagic protein Beclin 1-dependent autophagy through the interaction with Beclin 1 [39]. So *E. adenophorum* activated Beclin 1 and suppressed Bcl-2, resulting in the execution of apoptotic cell death via suppressing Bcl-2 family proteins according to the provious study [40, 41]. Also, the genetic manipulation of autophagy-mediated pathway indicates that the autophagy might be a protagonist of apoptosis. The results of this study proved that both autophagy and apoptosis were triggered by *E. adenophorum*. Considering this, we suspect that the excess activation of autophagy which is undergoing the unchecked degradative processes might contribute to the apoptosis in hepatocytes. In addition, the activation of the autophagy exceeds a certain threshold incuring the collapse of cellular function, which contributing to the apoptosis in hepatocytes. So the autophagy triggered by *E. adenophorum* performed cytotoxic functions of hepatocytes and autophagy preceded apoptosis. The PI3K/Akt/mTOR-mediated pathway is reported to be frequently activated in apoptosis and autophagy, and the inhibition of PI3K and Akt could cause significant cell apoptosis [42, 43]. So our results indicated that the apoptosis and autophagy of hepatocytes might be activated by *E. adenophorum* via the inhibition of PI3K, Akt and the suppression of PI3K/Akt/mTOR pathway. However, there are still many another common regulators affect both autophagy and apoptosis such as p53, protein c-FLIP (cellular FLICE-like inhibitory protein) [44]. So further study will be performed to explore the mechanism of the association between auophagy and apoptosis.

In summary, *E. adenophorum* caused the induction of apoptosis of apoptosis through the activation of mitochondrial pathway. In addition, the activation of hepatocellular autophagy was triggered by *E. adenophorum* via activating PTEN and AMPK, and the inhibition of PI3K/Akt/mTOR pathway. These data provides a new clue to understand the underlying mechanisms of *E. adenophorum*-caused hepatotoxicity in Saanen goat. Obviously, there are still much for us to unveil the complete mechanisms underlying *E. adenophorum*-caused growth inhibition and hepatocytes apoptosis.

## MATERIALS AND METHODS

### Ethics statement

All experimental procedures with goats and animal care used in the present study had been given prior approval by the recommendations in the Guide for Sichuan Agricultural University Animal Care and Use Committee, Sichuan Agricultural University, Sichuan, China under permit NO. DKY-B20100805.

### Experimental animals and plant materials

A total of 16 saanen goats (average weight and age were 25.34 ± 1.11 kg and 3.15 ± 0.13 months) randomly selected as test samples were divided into four groups. Saanen goats of control, groups I, II and III were administrated with feedstuffs containing 0%, 40% (i.e. 400 g/kg), 60% (i.e. 600 g/kg), 80% (i.e. 800 g/kg) *E. adenophorum* for 3 months depending on previous study [45]. The saanen goats were fed 1kg feedstuffs per day. *E. adenophorum* leaves were dried and collected after washed, grinded and sieved at room temperature to generate dry powder for the experiment.

### Annexin-V/PI apoptosis detection by Flow Cytometry (FCM)

The livers were immediately removed and minced using scissors to form a cell suspension that was filtered through a 300-mesh nylon screen, washed twice with cold PBS (Phosphate Buffered Saline), and then suspended in cells in 1× binding buffer at a concentration of 1 × 10^6^ cells/ mL. Transfer 100 μL of the solution to a 5-mL culture tube, and then add 5 μL of Annexin V-FITC and 5 μL of Propidium Iodide (PI). Gently vortex the cells and incubate for 15 min at RT (25°C) in the dark. Add 400 μL of 1× binding buffer to each tube and analyze by flow cytometry (BD FACSCalibur, US) within 1 h.

### Real-time RT-PCR (qRT-PCR) analysis

Total RNA was isolated from the powder of liver (50 mg) using Trizol (Aidlab, China). Synthesis of single-stranded cDNA from 5 μg of RNA was performed according to the “TUREscript 1st strand cDNA Synthesis Kit” (Aidlab, China), the mRNA was reverse transcribed into cDNA. The cDNA was used as a template for qRT-PCR analysis. The β-actin gene was used as an endogenous control [25]. The primer sequences were: CCTGCTTCTTTCTTCATCGG (forward), AGGTGCCTGGACTCTTGGGT (reverse) for Bax; GGCTGGGATGCTTTGTG (forward), GAGCAGTGCCTTCAGAGACAGC (reverse) for Bcl-2; GCAGCAAACCTCAGGGAAAC (forward), GGTTTCC CTGAGGTTTGCTG (reverse) for Caspase-3; AAGAA CGAGCCTCAGTAATC (forward), GGATTACTGAGG CTCGTTCT (reverse) for Caspase-8; GAAGACCAGCAG ACAAGC (forward), TGAATCCTCCAGAACCAA (reverse) for Caspase-9; CCTGCTTCTTTCTTCATCGG (forward), AGGTGCCTGGACTCTTGGGT (reverse) for β-actin. The gene expression fold changes were calculated using cycle time(Ct) values [47].

### Western-blot analysis

Hepatocytes were harvested and washed with ice-cold PBS, then lysed with ice-cold RIPA lysis buffer (Beyotime Inst. Biotech, Beijing, China) with 1 mmol/L PMSF. Protein concentrations were calculated by BCA assay kits (Pierce, Rockford, IL, US). 20 μg of total cellular protein was subjected to 12% SDS-PAGE and transferred to PVDF membranes (Millipore, Atlanta, GA, US). The membranes were blocked with 5% defatted milk powder at room temperature for 1 hr and then immunoblotting was performed with primary antibodies at 4°C overnight, followed by HRP-conjugated secondary antibody at room temperature for 1 h. Following each step, the membranes were washed five times with PBS-T (PBS-Twen 20) for 3 min. Finally, the blots were developed using the enhanced chemiluminescence (ECL) system. Antibodies for Beclin-1, LC3, caspase-3, -8, -9, Bid, p-Akt, Akt, PTEN, p-AMPK, AMPK, p-PI3K, PI3K, p-mTOR, mTOR and others were obtained from Cell Signaling Technology (Dancers, Mass, USA) and the dilution of all antibodies was 1:1000 according to the instructions of Cell Signaling Technology (Dancers, Mass, USA).

### Caspase activity measurement

Caspases activities were measured by colorimetric assay kits (BioVision, Inc., Mountain View, California, US) according to the manufacture's recommendations. Briefly, cells from each group were lysed and protein concentrations were measured using BCA Protein Assay Reagent (Pierce, Rockford, IL, US). 200 μg of protein samples were incubated with each caspase substrate, respectively, at 37°C in a microplate for 4 h. Samples were then read at 405 nm in microplate spectrophotometer (BioTek Instruments, Inc., Winooski, US).

### DNA fragmentation assay

Both control and *E. adenophorum-*administration hepatocytes were collected and washed with PBS. DNA extraction was performed according to previous studies [48]. After dissolved in TE buffer, DNA was subjected to 2% agarose gel electrophoresis for DNA fragmentation analysis.

### Mitochondrial transmembrane potential (*ΔΨm*) assessment

The transmembrane potential *ΔΨm* was analysed using a 5,5′,6,6′-tetrachloro-1,1′,3,3′ tetraethylbenzi midazolcarbocyanine iodide (JC-1) Mitochondrial Potential Detection Kit (Biotium Inc., Hayward, CA, US). The cell suspension was filtered through a 300-mesh nylon mesh, washed twice with cold PBS and stained by JC-1in PBS for 15 min at room temperature in the dark, followed by flow cytometric analysis.

### 4′,6-diamidino-2-phenylindole (DAPI) and acridine orange/Ethidium bromide (AO/EB) staining

The livers were minced using scissors to form a cell suspension that was filtered through a 300-mesh nylon screen. For DAPI staining, the hepatocytes were fixed with 80% ethanol at room temperature for 30 min. The fixative was removed and the hepatocytes were washed with PBS for 3 times, and then incubated with DAPI (1 μg/ml) for 45 min at room temperature in the dark. For AO/EB staining, the cells without fixation were loaded with a 100 μl fresh-prepared AO/EB staining solution (100 μg/ml), then immediately observed under a Nikon fluorescence microscope (Nikon Inc., Japan) in less than 20 min.

### Monodansylcadaverine (MDC) staining of autophagic vacuoles

The livers minced using scissors to form a cell suspension that was filtered through a 300-mesh nylon screen. Autophagic vacuoles were labeled with 0.05 mmol/L MDC in PBS at 37°C for 10 min. Then, the cells were washed three times with PBS. Autophagic vacuoles in hepatocytes were observed under a fluorescence microscope (Olympus, BX-60, Japan). Fluorescence intensity of MDC was measured at an excitation wavelength of 380 nm, emission wavelength of 530 nm.

### Immunocytochemistry

The hepatocytes were fixed with 3% paraformaldehyde (PFA) for 15 min at 37°C. Next, permeabilization step was carried out with chilled methanol (100%) for 10 min at −20°C and then cells were incubated in a blocking solution containing 5% bovine serum albumin (BSA) and 1% Triton-X 100 for 1 h at 37°C. Cells were then incubated with the LC3-II antibody (Cell signal technology Inc., Beverly, MA, USA) for 12 h at 4°C followed by FITC-conjugated secondary antibody for 1 h at 37°C. Nuclei were stained with DAPI (1 μg/ml) for 10 min at 37°C. Fluorescence images were captured by LSM 700 confocal laser scanning microscope (Carl Zeiss, Oberkochen, Germany).

### TdT-mediated dUTP nick end labelling (TUNEL) assay

Hepatic tissue was fixed in 4% paraformaldehyde, embedded in paraffin and cut into 6 μm sections. TUNEL assay was conducted to study DNA fragmentation using the *in situ* cell death detection kit (Vazyme, Piscataway, NJ, USA) according to the manufacturer's instructions. After mounting the TUNEL positive cells, nuclei were counterstained with DAPI and the sections were observed at ×400 magnification under a Nikon microscope (Nikon Inc., Japan).

### Pathological observation

The livers were fixed in 4% buffered formaldehyde and routinely processed in paraffin. Thin sections (5 μm) of each tissue were sliced from each block and mounted on glass. Slides were stained with hematoxylin and eosin Y. Histological slides were examined on an Olympus light microscope.

### Transmission electron microscopy (TEM) observation

The ultrastructural morphology changes were observed under a transmission electron microscope. After *E. adenophorum* administration, the tissues were fixed with 3% glutaraldehyde, and post-fixed with 1% OsO4. Then samples were dehydrated in graded ethanol solutions, followed by embedment and section. Ultra-thin sections were stained with uranyl acetate and lead citrate, and then observed under a transmission electron microscope (JEM-1230, Tokyo, Japan) at 60 kV.

### Statistical analysis

All data are expressed as mean ± standard deviation (SD). Statistical analyses were performed to compare the experimental groups with the control group using a one-way analysis of variance (ANOVA) complemented with the Tukey-Kramer multiple comparison test with equal sample size. All the statistical analyses were performed using a commercially available statistical software package (SPSS15.0, SPSS Inc, USA).
